# Distinct neural mechanisms for action access and execution in the human brain: insights from an fMRI study

**DOI:** 10.1093/cercor/bhae163

**Published:** 2024-04-14

**Authors:** Giorgio Papitto, Angela D Friederici, Emiliano Zaccarella

**Affiliations:** Max Planck Institute for Human Cognitive and Brain Sciences, Department of Neuropsychology, Stephanstraße 1a, 04103 Leipzig, Germany; International Max Planck Research School on Neuroscience of Communication: Function, Structure, and Plasticity (IMPRS NeuroCom), Stephanstraße 1a, 04103 Leipzig, Germany; Max Planck Institute for Human Cognitive and Brain Sciences, Department of Neuropsychology, Stephanstraße 1a, 04103 Leipzig, Germany; Max Planck Institute for Human Cognitive and Brain Sciences, Department of Neuropsychology, Stephanstraße 1a, 04103 Leipzig, Germany

**Keywords:** action representations, retrieval, execution, action categories

## Abstract

Goal-directed actions are fundamental to human behavior, whereby inner goals are achieved through mapping action representations to motor outputs. The left premotor cortex (BA6) and the posterior portion of Broca’s area (BA44) are two modulatory poles of the action system. However, how these regions support the representation-output mapping within the system is not yet understood. To address this, we conducted a finger-tapping functional magnetic resonance imaging experiment using action categories ranging from specific to general. Our study found distinct neural behaviors in BA44 and BA6 during action category processing and motor execution. During access of action categories, activity in a posterior portion of BA44 (pBA44) decreased linearly as action categories became less specific. Conversely, during motor execution, activity in BA6 increased linearly with less specific categories. These findings highlight the differential roles of pBA44 and BA6 in action processing. We suggest that pBA44 facilitates access to action categories by utilizing motor information from the behavioral context while the premotor cortex integrates motor information to execute the selected action. This finding enhances our understanding of the interplay between prefrontal cortical regions and premotor cortex in mapping action representation to motor execution and, more in general, of the cortical mechanisms underlying human behavior.

## Introduction

Actions comprise a broad spectrum of features we selectively employ to fulfill self-instantiated intentions or goals (Jeannerod 2006). As a core capacity of our cognitive control system, we continuously generate and maintain action representations that are relevant to our goal while suppressing those that are not (Koechlin 2016; Gratton et al. 2018). Action representations, therefore, serve as a critical bridge that enables the selection and execution of appropriate actions (Wood and Grafman 2003; Huey et al. 2006).

At the neural level, action representations have been strongly linked to the lateral prefrontal cortex (LPFC; Fletcher and Henson 2001; Koechlin et al. 2003; Badre 2008), which is organized hierarchically, following an anterior-to-posterior gradient (Badre and Nee 2018; Christoff and Gabrieli 2000; Koechlin et al. 2003; Nee and D’Esposito 2016). While prominent models attribute a different functional specificity to each region falling along the gradient (Stout 2010), two shared assumptions are consistently found: First, posterior LPFC regions are associated with forming simple stimulus-action mappings, while anterior regions are involved in creating and storing complex relationships among behavioral rules. Second, premotor cortex regions are engaged in selecting a motor response to be executed in response to a specific sensory input (Koechlin et al. 2003; Badre 2008; Taren et al. 2011).

Within the posterior regions of the LPFC, the neural involvement of Broca’s area—which includes Brodmann’s area (BA) 44 and BA45 in the left inferior frontal gyrus (IFG)—in accessing action representations is particularly relevant due to the existence of several competing neural hypotheses about the similarity between action and language processing in this area. This is due to the allegedly comparable sequential nature of both systems (Greenfield 1991; Rizzolatti and Arbib 1998; Fiebach and Schubotz 2006; Lametti and Mattar 2006; Fogassi and Ferrari 2007; Corballis 2010; Fitch and Martins 2014; Häberling et al. 2016; Zaccarella et al. 2021; Kemmerer 2022). Within Broca’s area, BA44 has been found to be involved in the initiation and termination of simple action sequences and in the transition from one sequence to the other (Koechlin and Jubault 2006). Furthermore, BA44 activity has been found to increase at lower levels of the behavioral hierarchy, according to a model focusing on level-structured features of behavior, i.e. the Hierarchical Error Representation (HER) model (Alexander and Brown 2018; see also Koechlin et al. 2003; Koechlin and Jubault 2006; Koechlin and Summerfield 2007).

At a finer resolution, recent data cast doubt on the hypothesis that BA44 has a homogeneous functional nature, but rather that anterior–posterior functional distinctions exist between the sub-regions of the area. This distinction implies the presence of anteriorly located sub-regions toward BA45, and more posterior sub-regions along BA6 (Clos et al. 2013). A coordinates-based meta-analysis supports the distinctiveness of the roles played by different sub-regions within BA44 on motor processing, which reported consistent convergences in left BA44 exclusively for action execution, imitation, and motor imagery tasks (Papitto et al. 2020). Furthermore, this convergence was localizable in the most posterior portion of left BA44 (pBA44). A more anterior sub-region (aBA44) was conversely linked to language processing (Clos et al. 2013) and, specifically, to the processing of basic syntactic operations (Zaccarella and Friederici 2017; Zaccarella et al. 2017).

In light of this evidence, it has recently been proposed that the pBA44 sub-region constitutes an essential node of a broader cognitive control network and may play a crucial role in accessing mental representations of actions guided by contextual information (Zaccarella et al. 2021). However, this hypothesis has never been addressed experimentally in any study to date. In particular, current research has not yet investigated the type of information implemented in an action representation and how the modulation of this information affects pBA44 activity. If pBA44 is modulated by information-based changes, this would contradict previous claims that the area is responsible for encoding and storing motor sequences as individual components (Clerget et al. 2011) or for encoding action–goal relations (Fazio et al. 2009). Furthermore, a functional dissociation between pBA44 and the premotor cortex has not been demonstrated. Although the premotor cortex is thought to be responsible for action selection processes (Tanji et al. 1996; Miller and Cohen 2001), no study has yet distinguished between processes associated with accessing a set of action representations and processes associated with selecting and executing the required sequence from that set.

To address these points, we developed a functional magnetic resonance imaging (fMRI) delayed-movement finger-tapping experiment in which we asked participants to access and then execute, with their right hand, actions from different categories of finger-tapping sequences, while lying in the scanner. Participants learned the sequences during a behavioral training session 1 day before the experiment. During the experiment, we presented them with four stimuli, each one coding one out of four categories of sequences: (i) a specific sequence (Specific level), (ii) two sequences sharing similar motor features (Sub-Rule level), (iii) four sequences sharing an abstract rule (Rule level), and (iv) eight sequences sharing no motor or abstract features (General level). The categories were used to modulate in a linear fashion the information accessible via a specific stimulus. This categorization reflects the concept of abstraction policy, wherein a policy involves the correlation between a given state, an action, and the expected result, and policy abstraction refers to the degree to which a rule dictates a specific action (Ranti et al. 2015). A first-order policy directly links a stimulus to a response (i.e. Specific level). A second-order policy is a more generalized rule that associates a stimulus with a first-order rule (i.e. Sub-Rule level), and this pattern can extend to higher orders as well (i.e. Rule and General level, where the General level represents the context of all possible sequences). As more levels of contextual contingency are introduced, each subsequent level of contingency provides a contextual framework for a more generalized category of lower-level policies (Badre and Nee 2018).

Furthermore, trials were split into two phases: the Category Processing phase, where participants could access the category of action representations signaled by the cue, and the Execution phase, where participants performed the finger-tapping task.

At a behavioral level, we hypothesized that the time participants required to start the finger-tapping sequence in the Execution phase would be influenced by the information carried by a particular category. Specifically, we hypothesized a step-wise increase in reaction time data for starting the tapping movement from Specific to General categories. On the other hand, we did not expect the transitioning time from one finger-tapping movement to the other (i.e. time from the first press until the second press and time from the second press until the third press) to be affected by the amount of information provided by the category. These hypotheses are informed by the fact that the time to perform the first finger-tapping movement of a sequence also reflects the planning phases of the execution task (Botwinick and Thompson 1966; Westerholz et al. 2013), while later stages occur after the motor plan is already formed.

At the neural level, we hypothesized that, if pBA44 is a crucial area for accessing action representations, activity within the area would be linearly modulated by the broadness of information carried by a particular stimulus or category. The more specific the information accessed when processing a category, the more activity should be observed in pBA44. This finding would shed light on the role played by the area, explicitly suggesting that pBA44 serves as a hub where action representations are retrieved according to the information they provide and that these representations are maintained until action execution is complete. However, while pBA44 selects motor information before the execution of the action (i.e. during the Category Processing phase), this information needs to be processed by the premotor cortex for performance (i.e. during the Execution phase). We hypothesized, therefore, a temporal and functional dissociation between pBA44 and BA6, with BA6 compensating for the integration of motor information from different trial phases. This modulation would result in a linear increase in premotor activity during the Execution phase for the more general action categories. These predictions are summarized as follows: (i) stronger activity in pBA44 during the Category Processing phase for more specific categories and (ii) stronger activity in BA6 during the Execution phase for more general categories.

## Materials and methods

### Participants

Data from 32 native German speakers (17 female; mean age = 26.91 years; standard deviation [SD] = 4.86; range = 18 to 36) were analyzed. Each participant took part in one training session and one fMRI session. A total of 42 participants were initially recruited. Still, one participant could not enter the magnetic resonance imaging (MRI) scanner because of a tattoo near the face area, two participants did not comply with the task during the fMRI session, and seven participants performed poorly during the training and were excluded from the study. All participants were right-handed (mean laterality quotient = 88.22; SD = 15.39), as assessed with the Edinburgh handedness test (Oldfield 1971), and had no history of neurological disorders or head injury or exhibited contraindications to fMRI. They were recruited via the participant database of the Max Planck Institute for Human Cognitive and Brain Sciences (MPI CBS), Leipzig, Germany. Written informed consent was obtained from each participant before the experiment. The study was performed according to the guidelines of the Declaration of Helsinki, and it was approved by the local ethics committee of the University of Leipzig. All participants received monetary compensation after completing the experiment: they were reimbursed 9€ per hour for participating in the training session and 10€ per hour for participating in the fMRI session. In addition, each participant could earn a little additional monetary compensation depending on their performance (as specified in Sections fMRI session and Behavioral training).

### Experimental procedure

**Fig. 1:**
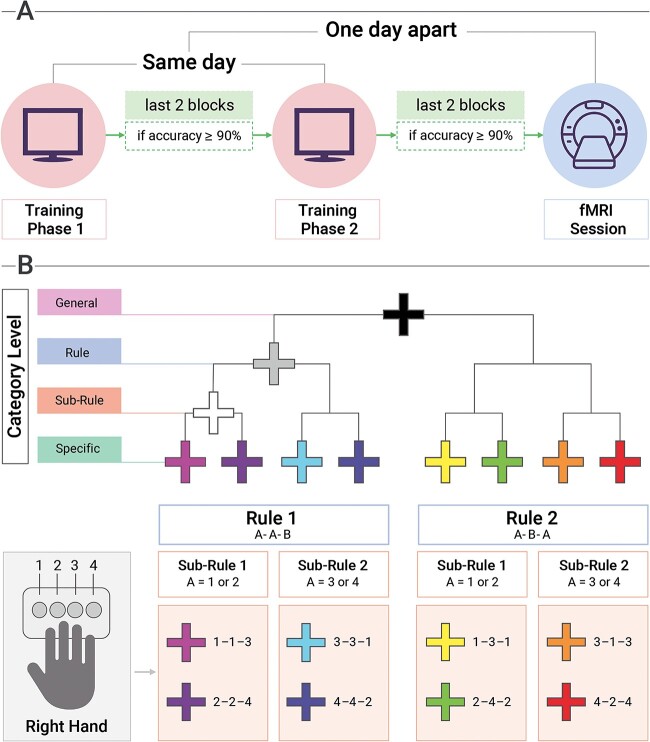
Experimental procedure and stimuli. Participants underwent both a behavioral Training Session and an fMRI Session. A) The Training Session comprised two different Training Phases, Training Phase 1 (TP1) and Training Phase 2 (TP2), which took place on the same day. To access TP2, participants’ accuracy in the last two blocks of TP1 had to be equal to or higher than 90%. To access the fMRI session, participants’ accuracy in the last two blocks of TP2 had to be equal to or higher than 90%. The fMRI Session took place 1 day after the Training Session. During the Training Session and fMRI Session, participants were presented with various cues, which could be assigned to different categories. B) The hierarchical categorization of cues into categories featured (i) a black fixation cross coding all possible sequences (General level); (ii) a gray fixation cross coding sequences following a specific pattern (i.e. Rule level); (iii) a white fixation cross coding sequences following a specific pattern and starting with a specific finger (i.e. Sub-Rule level); (iv) each colored fixation cross codes one specific finger-tapping sequence (i.e. Specific level). Sequences pertaining to the Rule level could follow one of these patterns: A-A-B (e.g. 1-1-3) or A-B-A (e.g. 1-3-1). Each number corresponds to a finger of the right hand (“1” for the index finger, “2” for the middle finger, “3” for the ring finger, and “4” for the little finger), and each finger is associated with a button of the button-box. Sequences pertaining to the Sub-Rule level were classified according to what finger corresponded to element A of the sequence. As such, in the sequences of Sub-Rule 1, the element A could be either the index finger (i.e. 1) or the middle finger (i.e. 2). Conversely, in the sequences of Sub-Rule 2, the element A could be either the ring finger (i.e. 3) or the little finger (i.e. 4). The hand icon was created with BioRender.com.

The study consisted of an fMRI session (Section fMRI session), preceded by a 1-day behavioral training session (Section Behavioral training) to get participants acquainted with the experimental task (for a schematic representation of the experimental procedure, see Fig. 1A). A behavioral pilot experiment was performed before the actual fMRI experiment to validate the task (see Supplementary Section 1.1).

#### fMRI session

Participants executed unimanual (right hand only) finger-tapping movements in response to visual cues. To avoid uncontrolled visual stimulation by the sight of their own hands and the button-box and systematic eye movements toward the button-box, we scanned participants in a standard fMRI configuration (i.e. horizontally, without tilting the head toward the body). We also instructed them to maintain fixation on the center of the screen throughout the experiment. These measures prevented direct viewing of their limbs and the button-box. A magnetic resonance-compatible response button-box for the right hand was used throughout the experiment. The button-box had four buttons (index to little finger), and, to ensure a comfortable setting, we adjusted its position individually to match each participant’s arm’s length. While in the scanner, eye-tracking data (fixation and pupil size measures) were recorded using the EyeLink 1000 Plus (SR Research Ltd, Ottawa, Ontario, Canada), a high-quality video eye-tracker compatible with MRI settings. Eye-tracking data were collected for research purposes unrelated to the current study and will not be discussed further. Stimulus presentation, response collection, and synchronization with the scanner were controlled using PsychoPy (version 2021.2.3; Peirce 2007, 2009).

The day before the fMRI session, each participant underwent a training session outside the scanner (for details on the training session, see Section Behavioral training). In the training session, participants learned to perform eight finger-tapping sequences. Each sequence was associated with a colored fixation cross and was presented to the participant in numbers. Each number corresponds to a specific finger of the right hand: “1” for the index finger, “2” for the middle finger, “3” for the ring finger, and “4” for the little finger. Each finger-tapping sequence consisted of three movements, and it involved only two fingers. Sequences could then be split into smaller categories, following the principle of abstraction policy. The eight sequences of the General level could be split into two groups of the same number of sequences, according to the rule (R) they followed. The rules were: (R1) all sequences of the first group start with a repetition of the same finger, following an A-A-B structure (i.e. “1-1-3”; “2-2-4”; “3-3-1”; “4-4-2”); (R2) all sequences of the second group start and end with the same finger, following an A-B-A structure (i.e. “1-3-1”; “2-4-2”; “3-1-3”; “4-2-4”). The sequences of R1 and R2 groups could be split into two further sub-groups containing the same number of sequences, according to the sub-rule (S) they followed. The sub-rules were: (S1) all sequences of the first sub-group start with either the index or middle finger (e.g. “1-1-3”) and (S2) all sequences of the second sub-group start with either the ring or the little finger (e.g. “4-4-2”). Such a specification produces the following four groups of sequences, according to the combination of rule and sub-rule: R1S1 (“1-1-3”, “2-2-4”), R1S2 (“3-3-1”, “4-4-2”), R2S1 (“1-3-1”, “2-4-2”), and R2S2 (“3-1-3”, “4–2-4”). Linking this schematization to policy abstraction, the General level encodes eight sequences out of an infinite number of behaviors. These sequences are competing with each other, as they are simultaneously available for execution. At the Rule level, the competition is cut by half leaving four possible sequences out of the initial eight (e.g. sequences following the A-A-B structure). The Sub-Rule level encodes only a sub-set of those sequences (e.g. A-A-B sequences starting with either the index or middle finger). At the Specific level, the number of simultaneously possible sequences is reduced to one. Therefore, each level represents a linear increment in abstraction policy: Sequence (first-order policy), Sub-Rule (second-order policy), Rule (third-order policy), and General (fourth-order policy; Sayalı et al. 2023).

Three additional cues experimentally introduced this distinction into Rules and Sub-Rules, each cue coding a (sub-)group: (i) one white fixation cross coding two sequences sharing a sub-rule (e.g. R1S1), (ii) one gray fixation cross coding four sequences sharing an abstract rule (e.g. R1), and (iii) one black fixation cross coding all the eight possible sequences. We used four sets of category-sequences assignments balanced across participants (four groups of participants, each group composed of eight participants). To make an example, this was the hierarchical categorization and the cues learned by Group A: (i) the black fixation cross codes all possible sequences; (ii) the gray fixation cross codes the sequences having an A-A-B structure (i.e. R1); (iii) the white fixation cross codes the sequences having a repetition and starting with either the index or the middle finger (i.e. R1S1); and (iv) each colored fixation cross codes one specific finger-tapping sequence (e.g. the pink fixation cross codes the sequence 1-1-3). For the Sequence cues, we used the following colors: pink, purple, light blue, blue, orange, red, yellow, and light green. To prevent differences in brightness, we used the same saturation (*S* = 100%) and luminance (*L* = 50%) levels for the colored cues, as featured in the HSL color space. Each category represents a level of the experimental condition Category: (i) General; (ii) Rule; (iii) Sub-Rule; and (iv) Sequence (for a schematic representation of the stimuli, see Fig. 1B). Participants were given detailed instructions on the specific requirements of the task before entering the scanner. In addition, they were provided with a printed slideshow containing task instructions, a list of the sequences, and a schema of their categorization.

**Fig. 2:**
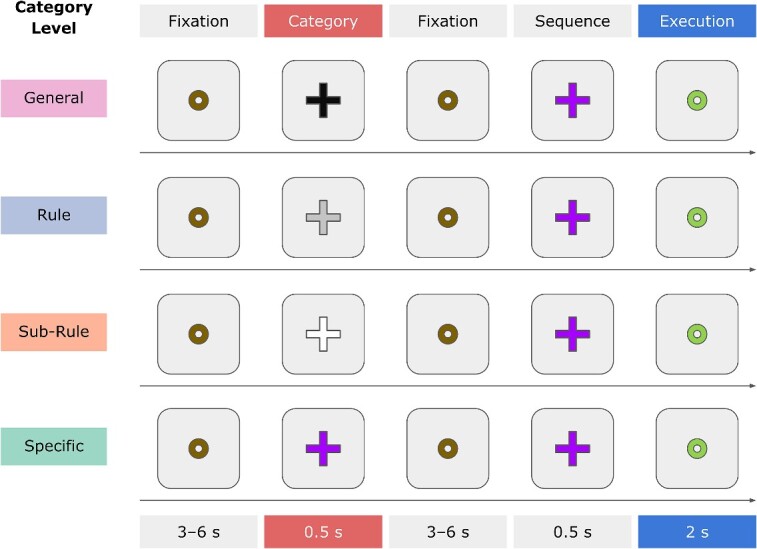
Example experimental trials per Category level. Each trial started with a fixation circle (Fixation cue) lasting for a variable amount of time, which served to alert participants of the upcoming trial. The duration of the fixation circle was randomly chosen between 3,000 and 6,000 ms, in steps of 500 ms. The fixation circle was followed by a colored cross (Category cue) for 500 ms, indicating one of the four action categories (i.e. Specific, Sub-Rule, Rule, or General). The colored cross was followed by a jittered inter-stimulus interval, during which the Fixation cue was shown again between 3,000 and 6,000 ms, in steps of 500 ms. At the end of the delay period, an additional colored cross (Sequence cue) was shown for 500 ms, indicating the finger-tapping sequence to execute. Finally, the Execution cue shown for 2,000 ms provided the GO-cue to start the movement and to return to the home position after completing the movement.

To temporally isolate neural processes pertaining to category processing from those associated with movement execution, we used a delayed-movement paradigm (Gallivan et al. 2011a; Gallivan et al. 2011b; Ariani et al. 2015, 2022). In particular, our design was an adaptation of the delayed-movement paradigm employed to test the processing of motor plans (Ariani et al. 2015). Each trial started with a brown fixation circle lasting for a variable time (Rest phase), which served to alert participants of the upcoming trial. The duration of the fixation circle was randomly chosen between 3,000 and 6,000 ms, in steps of 500 ms, with one limitation: the sum of the duration of fixation circles could not exceed 16 min. The fixation circle was followed by a colored cross (Category cue) for 500 ms (Category Processing phase), indicating one of the four action categories (i.e. Specific, Sub-Rule, Rule, or General). The colored cross was followed by a jittered inter-stimulus interval (ISI phase), during which the brown fixation circle was shown again between 3,000 and 6,000 ms, in steps of 500 ms, with the same limitation as the previous fixation circle. At the end of the delay period, an additional colored cross (Sequence cue) was shown for 500 ms, indicating the finger-tapping sequence to execute (Sequence Processing phase). Finally, a dark green circle provided the GO-cue to start the movement (Execution phase, 2,000 ms) and to return to the home position after completing the movement. Participants were asked to keep their hand still and relaxed on the button-box throughout all trial phases. In the original paradigm by Ariani et al. (2015), the duration of the Execution phase was 2,500 ms. We decided to shorten it, given the average reaction times we observed in our behavioral pilot study (see Supplementary Section 1.2). For a schematic representation of a trial for each Category level, see Fig. 2.

Each participant was shown a total of 228 trials for a total testing time of ca. 48 min. Trials were organized into six blocks, with the same number of trials in each block. Therefore, each block comprised 38 trials (6 of which were filler trials). The 32 experimental trials were split into equal amounts across conditions (8 trials per condition per block). Filler trials were defined as trials in which the relationship between the Category cue and the Sequence cue was wrong (e.g. participants were shown the cue coding R1S1 followed by the presentation of a sequence belonging to R2). In filler trials, participants had to refrain from any movement and wait for the next trial. Trials were presented pseudo-randomly with the following restrictions: (i) The category shown in the Category Processing phase could be repeated a maximum of two times in succession; (ii) the sequence shown in the Sequence Processing phase could be repeated a maximum of two times in succession; and (iii) a filler trial could not be immediately followed by another filler trial. After each block, a more extended rest period (24 s) allowed participants to relax their right arm, wrist, and hand. During this rest period, participants were shown a brown fixation square, identical in color to the fixation circle. To keep motivation high, participants were rewarded based on their performance, measured in terms of accuracy. If participants had an accuracy higher than 90%, they were granted a little additional monetary compensation.

#### Behavioral training

To ensure participants could learn the finger-tapping sequences and perform the tasks accurately, all participants participated in a behavioral training session outside the scanner the day before the experimental (scanning) session. The training session consisted of two phases: Training Phase 1 (TP1) and Training Phase 2 (TP2). Before starting the training, the specific requirements of both phases and the tasks were described to participants in detail. They were provided a printed slideshow containing all the necessary information, including the sequences and their categorization. Between TP1 and TP2, there was a break of 15 min to let participants rest. During the break, participants could also read the printed slideshow once more. The overall duration of the training session was ca. 120 min. During TP1 and TP2, participants were seated in front of a computer screen and provided with a button-box (16 × 8 cm) for the right hand. The button-box had four buttons (index to little finger) and was placed about 30 cm from the participant. The monitor was placed on the table (78 × 78 cm) behind the button-box. The distance between the eyes of the participant and the monitor was approximately 60 cm. The distance between the monitor and the button-box was about 16 cm.

TP1 and TP2 were both organized in six blocks of trials. However, to motivate participants to perform well already in the first blocks of each training phase, they were told that the number of blocks could vary between four and eight. Each block comprised 38 trials (6 of which were filler trials). Filler trials were trials in which the relationship between the Category and Sequence cues was wrong, as in the fMRI session. The 32 experimental trials were split into equal amounts across conditions (8 trials per condition per block). Between the blocks, there was always a break, lasting a maximum of 120 s. At the end of each block, participants were given feedback on how many trials they performed correctly. Stimulus presentation and response collection were controlled using PsychoPy (2020.2.10; Peirce, 2007) on a Linux workstation. To maintain participants’ engagement, a little monetary compensation was provided based on the participants’ performance in terms of accuracy.

During TP1, participants were presented with the following: (i) a brown fixation circle; (ii) a Category cue; (iii) a second brown fixation cue; (iv) a Sequence cue; (v) a relation question; (vi) written feedback concerning the relation question; (vii) the sequence to be executed, in numbers; (viii) the Execution green cue; and (ix) written feedback concerning the finger-tapping performance. The fixation cues could last 500, 1,000, or 1,500 ms, and the duration value was randomly assigned. The Category and the Sequence cues were presented for 500 ms each. The relation question was shown for maximally 1,500 ms until participants responded, and it read as follows: “Is the sequence cue coded by the category cue?”. Participants could reply “Yes" or “No″ by pressing the first or last button on the button-box, respectively. This question was introduced so that participants could prove to have understood the categorical relation among the cues. The feedback to the answer was shown for 500 ms. If participants made a mistake in answering the question, the trial would end and the subsequent trial would start immediately. If participants gave the correct answer, the trial would continue. After a correct response, the sequence to be pressed was shown in numbers for 1,500 ms. The Execution cue was then shown for maximally 2,500 ms until participants responded. While the Execution cue was on screen, participants had to execute the finger-tapping sequence. After the Sequence cue, written feedback was shown on the screen for 2,000 ms. Only participants that reached at least 90% accuracy in the last two blocks of TP1 advanced to TP2 and earned a little additional monetary compensation.

The design of TP2 closely resembled the experimental session that participants would complete the next day, but with some key differences: (i) Feedback was provided after each trial in TP2, while no feedback was given during the fMRI session; (ii) fixation cues in TP2 could last between 500 and 2,000 ms (in steps of 500 ms), and the value of the duration was randomly assigned, while these could last between 3,000 and 6,000 ms (also in steps of 500 ms) in the fMRI session; (iii) the Execution cue in TP2 lasted maximally 2,500 ms, while it lasted maximally 2,000 ms in the fMRI session; and (iv) a trial in TP2 started immediately after all the three button presses of the finger-tapping sequence for the previous trial were completed or after 2,500 ms from the beginning of the Execution phase had passed, contrary to the fMRI session where a trial started exclusively after 2,000 ms from the beginning of the Execution phase of the previous trial had passed. Only participants who achieved at least 90% accuracy in the last two blocks of TP2 were eligible to move on to the fMRI session and received a little additional monetary compensation.

### Behavioral data acquisition

We acquired reaction times for each finger-tapping movement as follows: (i) time from the start of the Execution phase to the time the participant pressed the first button (P1); (ii) time from the release of P1 to the time the participant pressed the second button (P2); and (iii) time from the release of P2 to the time the participant pressed the third button (P3). Additionally, overall accuracy rates were also acquired for each Category level.

### fMRI data acquisition

fMRI data were collected on a 3T Skyra scanner (Siemens, Erlangen, Germany) using a 32-channel head coil. Functional, blood oxygenation level-dependent (BOLD) images were acquired using a T2*-weighted multiband gradient-echo echo-planar-imaging (EPI) sequence, with the following parameters: TR = 2,000 ms, TE = 22 ms, flip angle = 80°, FOV = 204, voxel size = 2.5 × 2.5 × 2.5 mm, interslice gap = 1 mm, multiband acceleration factor = 3, bandwidth = 1,966 Hz/Px, phase encoding direction = A/P. A total of 60 slices covering the whole brain were recorded in interleaved order and axial orientation. Structural T1-weighted images were previously acquired and retrieved from the institute brain database for all participants. Images were acquired using an MPRAGE sequence, with the following parameters: TR = 2,300 ms, TE = 2.98 ms, flip angle = 9°, FOV = 256 × 240 mm, voxel size = 1 × 1 × 1 mm, no interslice gap, phase encoding direction = A/P. A total of 176 slices covering the whole brain were recorded.

### Behavioral data analysis

Behavioral data acquired in the behavioral pilot and fMRI experiments were analyzed following the same procedure. Mean reaction times were calculated for each participant and Category level as the time needed to execute the different movements required in the Execution phase. In this regard, reaction time values were measured accordingly: (i) time from the start of the Execution phase to the time the participant pressed the first button (P1); (ii) time from the release of P1 to the time the participant pressed the second button (P2); and (iii) time from the release of P2 to the time the participant pressed the third button (P3). Before calculating the mean reaction times, we preprocessed the data for each participant separately. All data cleaning and preprocessing steps were conducted in Python (3.8), using Pandas (1.1.3) and NumPy (1.19.2) packages (McKinney 2011; Harris et al. 2020).

First, incorrect and missed trials and filler trials were removed. Second, outliers for each participant were removed. Outliers were defined as values 1.5 interquartile range (IQR) below the first quartile (Q1) and 1.5 IQR above the third quartile (Q3) (Tukey 1977). Hence, the lower inner fence was defined as Q1 to 1.5*IQR, while the upper inner fence was Q3 + 1.5*IQR. Subsequently, in the case data were not normally distributed, the PowerTransformer class from Python’s Scikit-learn (0.23.2) library was used to transform the values to a normal distribution-like representation using Yeo-Johnson’s transformation (Pedregosa et al. 2011). Normality was tested using the D’Agostino–Pearson normality test, as implemented in the scipy.stats (1.7.3) module of SciPy (Virtanen et al. 2020). Here, *P* < 0.05 indicates a non-normal distribution of reaction time values.

Mean P1, P2, and P3 values were then entered separately and analyzed into a one-way within-subject analysis of variance (ANOVA) with P1, P2, or P3 as dependent variables, and Category (four levels: Specific, Sub-Rule, Rule, General) as fixed factors. Given our hypothesis, paired-samples *t*-tests were performed as post-hoc comparisons exclusively for P1. In this case, since we provided a directionality hypothesis of our experimental manipulation, we applied one-tailed *t*-tests to compare the following level-to-level decreases: (i) Specific < Sub-Rule, (ii) Sub-Rule < Rule, and (iii) Rule < General mean values. All *P*-values obtained in the *t*-tests have been Bonferroni-corrected (Bland and Altman 1995), multiplying original values by the number of planned comparisons (i.e. 3). Only resulting values smaller than the 0.05 threshold were considered significant. ANOVAs and post-hoc analyses were conducted using the Pingouin (0.5.0) package (Vallat 2018).

To further support the evidence for the role played by the Category levels in P1 and against it for P2 and P3, we performed Bayesian statistical analyses applying this framework to each of the previously described statistical tests (i.e. one-way ANOVAs for P1, P2, and P3, and one-sided post-hoc comparisons across Category levels for P1). By describing how informative the data from a given experiment are, Bayesian analysis allows for quantifying evidence for both the null (H_0_) and the alternative (H_1_) hypotheses (Dienes 2014; Keysers et al. 2020). Within this statistical framework, the Bayes factors (BFs) indicate how likely the data are under these two hypotheses. For example, a BF_10_ equal to 5 means that the current data are five times more probable under H_1_ than H_0_. What is reported as BF_01_ is equivalent to 1/BF_10_, and it indicates how many times the data are more likely under H_0_ (Maran et al. 2022). The BF interpretation is guided by the previously defined benchmarks (Kass and Raftery 1995): BF < 3 indicates weak or anecdotal evidence, 3 ≤ BF < 20 indicates positive evidence, 20 ≤ BF < 150 indicates strong evidence, and BF > 150 indicates robust evidence. Bayesian analyses were performed using the JASP (0.16.3) software (JASP Team 2022). For all Bayesian paired-sample *t*-tests, we set the Cauchy prior width to JASP default value *r* = 0.707 (Wagenmakers et al. 2018a; Wagenmakers et al. 2018b).

Additionally, linear trend analyses were applied to P1, P2, and P3 mean values to estimate the negative or positive linear relation between independent (i.e. Category) and dependent (i.e. mean reaction time) variables (Pinhas et al. 2012). The traditional linear regression analysis only provides the slope to capture the essence of an effect. The advantage of the linear trend analysis here adopted is that it quantifies the effect size of both the slope and the proportion of variability accounted for (Aleotti et al. 2023).

Finally, given the inappropriateness of ANOVAs over categorical data (Jaeger 2008; Song et al. 2017), we employed generalized linear mixed models (GLMMs) of logistic regression to analyze accuracy data (Song et al. 2017; Park et al. 2019; Hoffman et al. 2020). Therefore, the GLMM with a binomial distribution and a logit link function was conducted to fit binary responses (0 = incorrect response, 1 = correct response) on each trial, using the Laplace approximation of the maximum-likelihood. This analysis was conducted in RStudio (RStudio Inc., Boston, MA, USA), using the lme4 (1.1-14) package (http://lme4.r-forge.r-project.org). To compute this analysis, we calculated a model including binary accuracy measures (dependent variable), one fixed effect variable (Category), and one random effect variable (participants). Model estimation was performed using the glmer function. This model was compared against the null (intercept-only) model to obtain significance measures. Finally, a model comparison was performed using the anova function (Zhu et al. 2021).

### fMRI preprocessing and analysis

#### Preprocessing

Functional data were analyzed using the SPM12 software package (Wellcome Trust Centre for Neuroimaging; http://www.fil.ion.ucl.ac.uk/spm/) implemented in MATLAB (version 2022b; Mathworks, Inc., Sherborn, MA, USA). Participant-specific functional volumes were coregistered in the preprocessing stage with corresponding structural T1-weighted images. Functional time series were further realigned to the first EPI image to correct for head motion artifacts, unwrapped to correct for geometric distortions due to susceptibility gradients based on the B0 field-map, and resliced for time series. We performed the normalization to the standard Montreal Neurological Institute (MNI) template as included in the SPM software package, and we used a Gaussian kernel of 5mm^3^ full width at half-maximum (FWHM) to smooth the data. We applied a high-pass filter with a cutoff period of 128 s to avoid low-frequency drift. The first three let-in volumes were excluded to allow for the magnetic saturation effect.

#### fMRI whole-brain data analysis

We estimated a general linear model (GLM) for each participant (Friston et al. 1994) and phase of interest (Category Processing and Execution), as implemented in SPM12. The model included one regressor for each condition and was constructed by convolving the onset and either the duration of stimulus presentation (for the Category Processing phase) or the total execution time (for the Execution phase). For both phases, filler trials were modeled as distinct conditions, and movement parameters were treated as regressors of no interest. For the Execution phase only, error trials were modeled as separate conditions. Contrast estimates for the four experimental conditions (compared against the global mean) were obtained using first-level statistics. The contrast estimates were then used in a second-level within-subjects ANOVA to assess group contrasts. Statistical inferences were drawn at *P* < 0.05, with a Family-Wise Error (FWE) correction at the voxel level. Effects of interest were tested at the whole-brain level by linear contrasts, generating statistical parametric maps of *t*-values at the group level. Positive linear contrasts (activity increasing with Category levels: from Specific to General) were defined as [−3−1 + 1 + 3], and negative linear contrasts (activity decreasing with Category levels: from Specific to General) as [+3 + 1 −1 −3]. Linear contrasts reflect a continuous increase or decrease in activation with each higher or lower level within an experimental condition. As such, they indicate an efficient upregulation or downregulation of neural activity at increasing or decreasing task loads (Bauer et al. 2018; Brown et al. 2018; Annak et al. 2019). The MNI2TAL application from the Yale BioImage Suite Package (https://bioimagesuiteweb.github.io/webapp/mni2tal.html) was used to locate cortical activation peaks.

#### Mass overlap analysis in BA44

A recent meta-analytic functional connectivity-based parcellation (CBP) approach proposed a decomposition of left BA44 into five separate sub-regions called clusters (Cs). Two of these are located in its more posterior part (C1 and C4), another two in the more anterior part (C2 and C3), and a last one in the inferior frontal junction (C5; Clos et al. 2013). These clusters are considered to be functionally specialized (C1: language and working memory; C2: working memory, semantics, orthography, reading, and covert speech; C3: semantics, syntax, phonology, and overt as well as covert speech; C4: action and action imagination; C5: task switching, attention, cognitive control, and detection of behaviorally relevant events). To further address the functional specification of these different clusters, we ran a mass overlap analysis. This aimed to determine the cluster or clusters that exhibit the highest overlap with the BA44-spanning cluster identified in the [+3 + 1 −1 −3] contrast, which revealed areas showing an increase in BOLD response with more specific action categories during the Category Processing phase. The resulting image of the linear contrast was overlaid onto a standard MNI template (Colin27_T1_seg_MNI; www.brainmap.org/ale/Colin27_T1_seg_MNI.nii.gz) and then displayed using the Mango brain visualization software (4.0.1; http://ric.uthscsa.edu/mango/). Following an established procedure (Ishaque et al. 2017), within Mango: (i) we used the *Threshold to ROI* function to create a region of interest (ROI) around each of the clusters identified by the contrast, (ii) we confirmed the ROIs by visual inspection, (iii) we saved the ROI spanning BA44, and (iv) we calculated the volume in voxels of the ROI using the *ROI Statistics → Volume* functions. As a second step, the obtained ROI and all the clusters identified by Clos et al. (2013) were overlaid onto the previously mentioned standard MNI template. To obtain the mass overlap, we used (i) the *Create Overlay Logicals* tool to show areas of overlap between the two component images (i.e. C1∩ROI; C2∩ROI; C3∩ROI; C4∩ROI; C5∩ROI) and (ii) we calculated the overlaps’ volumes (in voxels) using the *Create stats of this region* tool from within the Logicals interface. Next, the relative overlap between the ROI spanning BA44 and each cluster was computed by dividing the number of voxels overlapping between a specific cluster-ROI combination (e.g. C1∩ROI) by the sum of the voxels overlapping for each C∩ROI combination (C1∩ROI + C2∩ROI + C3∩ROI + C4∩ROI + C5∩ROI). To obtain the percentage of overlap, we then multiplied each value by 100.

#### Signal change analysis

The extraction of signal change for each level of the Category condition in the different regions can provide additional information about (sub-)regional activation specificities (i.e. left BA44 vs. left BA6) and directionality of activation (activation vs. deactivation). To obtain peak activation coordinates for the two regions, we performed the following two analyses at the group level: (i) a *t*-contrast [+3 + 1 −1 −3] for the Category Processing phase, using an inclusive mask of left BA44 and (ii) a *t*-contrast [−3 −1 + 1 + 3] for the Execution phase, using an inclusive mask of left BA6. Both masks were extracted using the Wake Forest University (WFU)-Pickatlas tool (http://www.fmri.wfubmc.edu/cms/software) implemented in SPM12 (Lancaster et al. 1997; Maldjian et al. 2003; Hallam et al. 2015). We defined spherical ROIs with a 6 mm radius centered around these peak coordinates. Time-series data were extracted from these ROIs for each participant, and we calculated percent signal change via MarsBaR (0.44; https://marsbar-toolbox.github.io/) as signal intensity for each Category level. The significance level for the ROI was *P* < 0.05. Four separate one-way linear trend analyses were used to estimate the negative or positive linear relation between independent (i.e. Category) and dependent (i.e. percent signal change) variables (Pinhas et al. 2012). We tested for percent signal change’s linearity in the BA44 and BA6 ROIs during both the Category Processing and Execution phases. As such, the ROI analyses uses the following ROI × Phase configurations: (i) GLM-identified Category-Processing-related pBA44 to address pBA44 behavior during Execution; (ii) GLM-identified Execution-related BA6 to address BA6 behavior during Category Processing; (iii) GLM-identified Category-Processing-related pBA44 to address pBA44 behavior during Category Processing; and (iv) GLM-identified Execution-Processing-related BA6 to address BA6 behavior during Execution. As a disclaimer, we do not rely on results from analyses (iii) and (iv) for interpretation, but only on those from (i) and (ii) and/or from the GLM analysis (Section Signal change analysis).

### Preregistration

Before participants’ recruitment, we preregistered hypotheses and methods for the fMRI study through aspredicted.org (preregistration *#*85319; https://aspredicted.org/mb92p.pdf). In this section, we mention deviations from the preregistered methods. Firstly, to investigate the presence of a linear decrease in left BA44 during the Category Processing phase and a linear increase in left BA6 during the Execution phase, we preregistered two masked analyses, which would employ the respective ROIs as inclusive masks. However, we later reasoned that looking at the whole brain level would be more informative and unbiased (see Section fMRI whole-brain data analysis). Secondly, we decided to perform additional exploratory analyses. As a result, we performed the following exploratory nonpreregistered analyses: (i) GLLM on accuracy data (see Section Behavioral data analysis); (ii) mass overlap analysis (see Section Mass overlap analysis in BA44); and (iii) signal change analysis (see Section Signal change analysis).

## Results

In this section, we report the behavioral results of the fMRI session (Section fMRI session: behavioral results) and the fMRI results (Section fMRI results) in the following order: fMRI whole-brain results (Section fMRI whole-brain data analysis), mass overlap results (Section Mass overlap analysis in BA44), and signal change results (Section Signal change analysis). The behavioral results of the pilot experiment are reported in the Supplementary Materials (Supplementary Section 1.2).

### fMRI session: behavioral results

**Fig. 3:**
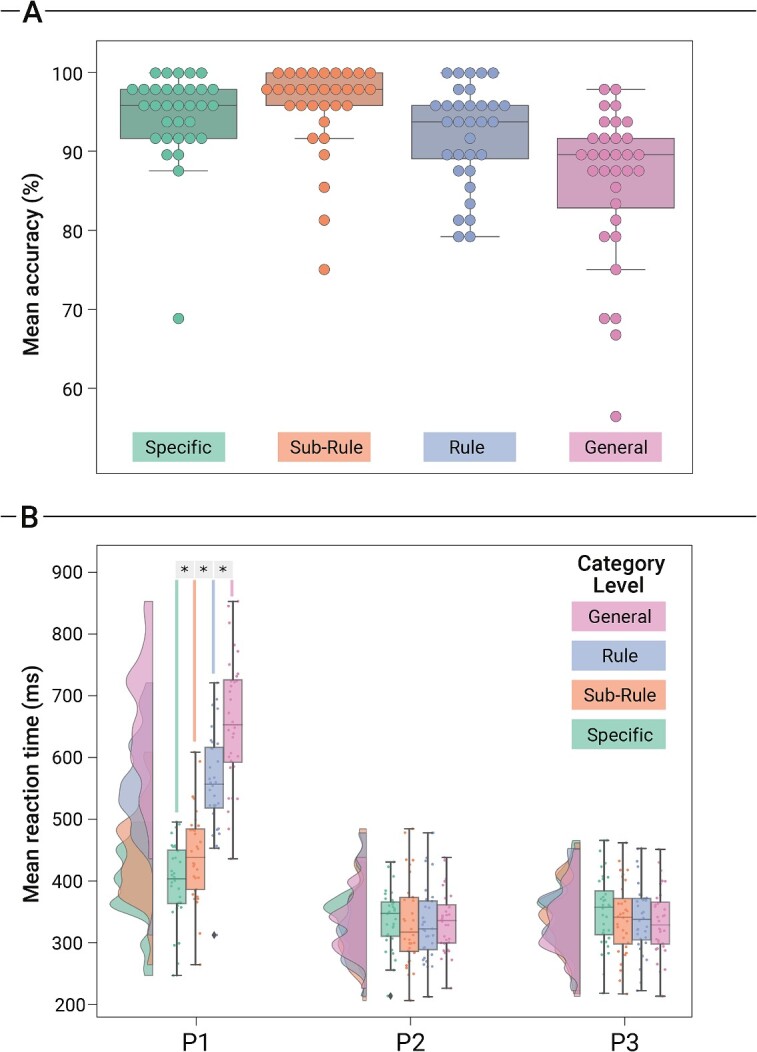
Behavioral results of the fMRI experiment. Boxplots A) indicate the mean percentage of accuracy values (%) for each Category level (Specific, Sub-Rule, Rule, General). Vertical bars indicate standard error means, the thick horizontal line inside the box indicates the group median, the bottom and top of the box indicate the group-level first and third quartiles of each Category level, and the colored dots represent the accuracy value for each participant. Raincloud plots B) show mean reaction time values in seconds (s) for each finger press (P1, P2, and P3) and for each Category level (Specific, Sub-Rule, Rule, General). Vertical bars indicate standard error means, the outer shapes represent the distribution of the data over participants, the thick horizontal line inside the box indicates the group median, the bottom and top of the box indicate the group level first and third quartiles of each Category level, the rhombus indicate outliers within the data, and the colored dots represent the mean value for each participant. Asterisks indicate statistically significant comparisons (*P* < 0.05).

Participants were generally accurate in performing the delayed-movement task (mean = 92.834%; SD = 5.214). Logistic regression analysis results show a main effect of Category on accuracy rates (*x*^2^_3_ = 116.74; *P* < 0.001), with more specific Category levels leading to higher accuracy rates. Mean accuracy was calculated for each level of the variable Category: *Specific* (mean = 94.661%; SD = 5.843), *Sub*-*Rule* (mean = 95.703%; SD = 5.771), *Rule* (mean = 92.188%; SD = 6.350), and *General* (mean = 86.068%; SD = 9.792; see Fig. 3A for an overall outlook of the accuracy data). Below, we report frequentist and Bayesian analyses for each finger-tapping movement (see Fig. 3B for an overall outlook of the reaction time data).

#### Press 1 (P1)

A frequentist one-way ANOVA on mean P1 values resulted in a main effect of Category [F_(3,124)_ = 63.909; *P* < 0.001]. Similar results were obtained when employing a Bayesian ANOVA, which attested robust evidence for rejecting the null hypothesis (BF_10_ = 6.906 + e9). To check for significant increases in P1 from one Category level to the other, we ran three one-sided paired-sample *t*-tests (Bonferroni corrected for three comparisons) as follows: (i) *Specific* < *Sub*-*Rule*; (ii) *Sub*-*Rule* < *Rule*; and (iii) *Rule* < *General*. Respectively, we observed that: (i) P1 values relative to the *Specific* level are reduced (mean = 0.398; SD = 0.063) when compared to those of the *Sub-Rule* level [mean = 0.441; SD = 0.074; *t*_(31)_ = −4.309; *P* < 0.001]; (ii) P1 values relative to the *Sub-Rule* level are reduced when compared to those of the *Rule* level [mean = 0.557; SD = 0.084; *t*_(31)_ = −12.073; *P* < 0.001]; and (iii) P1 values relative to the *Rule* level are reduced when compared to those of the *General* level [mean = 0.657; SD = 0.104; *t*_(31)_ = −9.435; *P* < 0.001]. In addition, paired-sample post-hoc comparisons were also performed using a Bayesian approach. Coherently with the results obtained employing a frequentist approach, we observed: (i) robust evidence in favor of the alternative hypothesis when testing for *Specific* < *Sub-Rule* (BF_−0_ = 346.872); (ii) robust evidence when testing for *Sub-Rule* < *Rule* (BF_−0_ = 5.129 + e10); and (iii) robust evidence when testing for *Rule* < *General* (BF_−0_ = 1.615 + e08). Finally, a linear trend analysis revealed that P1 values were associated with a significant positive linear trend [*F*_(1, 31)_   =  263.29; *P* < 0.001; *η^2^_p_*   = 0 .89].

#### Press 2 (P2)

A frequentist one-way ANOVA on mean P2 values resulted in no significant main effect of Category [F_(3,124)_ = 0.050; *P* = 0.985]. While the classical frequentist ANOVA rejected H_1_, a Bayesian ANOVA provided positive evidence in favor of H_0_ (BF_01_ = 22.600). The following mean values for each Category level were observed: (i) *Specific* (mean = 0.338; SD = 0.048); (ii) *Sub*-*Rule* (mean = 0.333; SD = 0.069); and (iii) *Rule* (mean = 0.333; SD = 0.064); and (iv) *General* (mean = 0.335; SD = 0.050). Finally, a linear trend analysis revealed for P2 values did not reveal a significant linear trend [*F*_(1, 31)_   = 0.99; *P* = 0.60; *η^2^_p_*   = 0 .03].

#### Press 3 (P3)

A frequentist one-way ANOVA on mean P3 values resulted in no significant main effect of Category [F_(3,124)_ = 0.453; *P* = 0.715]. While the classical frequentist ANOVA rejected H_1_, a Bayesian ANOVA provided weak evidence in favor of H_0_ (BF_01_ = 14.280). The following mean values for each Category level were observed: (i) *Specific* (mean = 0.350; SD = 0.057); (ii) *Sub*-*Rule* (mean = 0.339; SD = 0.059); (iii) *Rule* (mean = 0.339; SD = 0.057); and (iv) *General* (mean = 0.333; SD = 0.055). Finally, a linear trend analysis revealed that P3 values were associated with a significant negative linear trend [*F*_(1, 31)_   =  22.15; *P* < 0.001; *η^2^_p_*  =  0.42].

### fMRI results

#### fMRI whole-brain data analysis

**Fig. 4:**
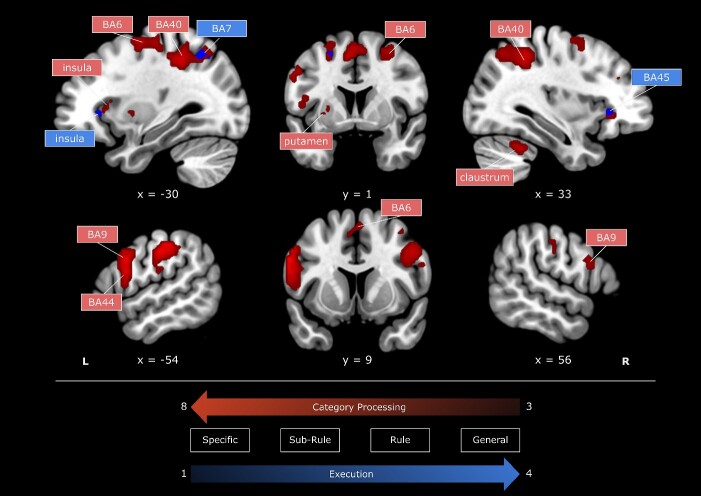
Whole-brain analysis. Regions of the brain that exhibited a negative linear response during the Category Processing phase (parametric *t*-contrast: “ + 3”: Specific; “+1”: Sub-rule; “−1”: Rule; “−3”: General; in red) or a positive linear response during the execution phase (parametric *t*-contrast: “−3”: Specific; “−1”: Sub-rule; “+1”: Rule; “+3”: General; in blue). Images were generated using MRIcroGL. All presented clusters are obtained after the *P* < 0.05 correction at the voxel level. The results are superimposed on the MNI template, and the numbers of the color bars correspond to *T*-values. BA = Brodmann area, L = left, R = right.

We performed a negative linear contrast to identify brain regions associated with different Category levels in the Category Processing phase [+3 + 1 −1 −3]. This contrast identifies brain regions that are the most engaged in the *Specific* level and decrease their activity as the specificity of the Category decreases (i.e. “ + 3”: Specific; “ + 1”: Sub-Rule; “−1”: Rule; “−3”: General). The biggest cluster was centered around the left postcentral gyrus (mainly in BA2 and marginally in BA3) and included the left inferior parietal lobule (BA40). A second large cluster was centered around the left precentral gyrus (BA6) and extended toward the IFG (BA44 and BA9), reaching the insula. Subcortically, additional left-hemispheric clusters comprised areas of the thalamus and putamen. The most widespread right-hemispheric cluster was centered around the right parietal cortex, extending from the inferior parietal lobule (BA40) to the postcentral gyrus (BA2) and the precuneus (BA7). A second cluster was centered around the right middle frontal gyrus (BA6) and reached the IFG (BA9). One smaller right-hemispheric cluster comprised the insula and marginal portions of the inferior frontal gyrus. Subcortically, right-hemispheric clusters contain areas of the culmen and the cerebellar tonsil as part of the cerebellum (see Fig. 4 and Supplementary Table 1).

To identify brain regions associated with different Category levels in the Execution phase, we performed a positive linear contrast [−3 −1 +1 +3]. Therefore, this contrast identifies brain regions that are the least engaged in the *Specific* level and increase their activity as the specificity of the Category decreases (i.e. “−3”: Specific; “−1”: Sub-Rule; “ +1”: Rule; “ +3”: General). One left-hemispheric cluster comprised the insula and, marginally, the IFG. A second left-hemispheric cluster was centered around the superior parietal lobule (BA7). Finally, a cluster in the left hemisphere was found in the sub-gyral portion of the left frontal lobe (BA6; see Fig. 4 and Supplementary Table 2).

#### Mass overlap analysis in BA44

**Fig. 5:**
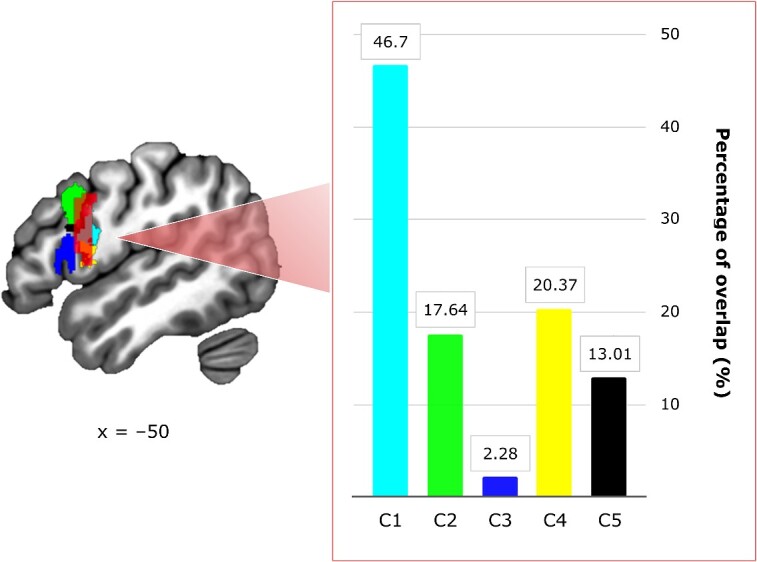
Mass overlap analysis. Overlap between the inferior-frontal cluster derived from the negative linear contrast [+3 +1 −1 −3] in the category processing phase and the functional clusters (Cs) of left BA44. The sagittal plane of the left hemisphere at *x* = −50 shows the total overlap of the negative linear contrast [+3 +1 −1 −3] in the category processing phase (in red) with each cluster (C1 in light blue, C2 in green, C3 in blue, C4 in yellow, C5 in black) of left BA44 identified by Clos et al. (2013). The following percentages of relative overlap (%) with each C were observed: C1 = 46.7% (in light blue), C2 = 17.64% (in green), C3 = 2.28% (in blue), C4 = 20.37% (in yellow), C5 = 13.01% (in black).

Clos et al. (2013) recently proposed a decomposition of left BA44 into five separate clusters (Cs). The clusters are considered to be functionally specialized (C1: language and working memory; C2: working memory, semantics, orthography, reading, and covert speech; C3: semantics, syntax, phonology, and overt as well as covert speech; C4: action and action imagination; C5: task switching, attention, cognitive control, and detection of behaviorally relevant events). To address the functional specification of the Cs of left BA44, we ran a mass overlap analysis to investigate with which C the BA44-spanning cluster observed for the [+3 +1 −1 −3] contrast at the whole brain level overlaps the most. We found that 58.22% of the inferior-frontal cluster observed for the negative linear contrast [+3 +1 −1 −3] in the Category Processing phase overlaps with the functional Cs of BA44. Of the total overlap, we observed that voxels of the inferior-frontal cluster fell mainly into the more posterior Cs of BA44 (C1 = 46.7%; C4 = 20.37%). Smaller overlap was observed with the anterior-dorsal cluster (C2 = 17.64%) and the cluster in the inferior frontal junction (C5 = 13.01%). Only marginal overlap was observed with the anterior-caudal cluster of BA44 (C3 = 2.28%; see Fig. 5 and Supplementary Table 3).

#### Signal change analysis

**Fig. 6:**
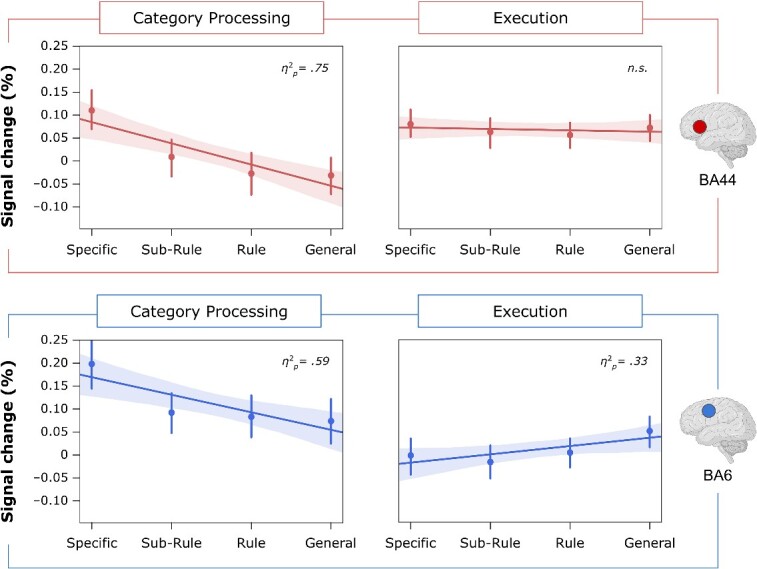
Signal change for each trial phase and ROI. Linear plots show linear regression analysis on the mean percentage of signal change (%) for each phase (Category Processing and Execution) and each ROI (left BA44 at the top and left BA6 at the bottom). The signal change was extracted with MarsBaR (Brett et al. 2002). Shaded areas correspond to the confidence interval bands of the linear fit, and vertical bars indicate standard error means. We report for each linear regression analysis the partial eta-squared (*η*^2^*_p_*) as an estimate of the magnitude of the linear component (Pinhas et al. 2012). Brain icons were created with BioRender.com.

Mean percent BOLD signal change values of BA6 and BA44 ROIs were submitted to four separate one-way linear trend analyses to estimate negative or positive linear relations between independent (i.e. Category) and dependent (i.e. percent signal change) variables. We tested for signal change’s linearity in the BA44 and BA6 ROIs during the Category Processing and Execution phases (see Fig. 6). During the Category Processing phase, BA44 revealed a significant negative linear trend [*F*_(1, 31)_   = 93.85; *P* < 0.001; *η^2^_p_* =  0.75]. No significant linear trend was observed for BA44 as a function of Category levels during the Execution phase [*F*_(1, 31)_   = 0.52; *P* = 0.30; *η^2^_p_*   = 0.02]. The analysis of BA6 signal change revealed a significant negative linear trend in the Category Processing phase [*F*_(1, 31)_  =  45.3; *P* < 0.001; *η^2^_p_*   = 0.59] and a significant positive linear trend in the Execution phase [*F*_(1, 31)_  =  15.1; *P* < 0.001; *η^2^_p_*   = 0 .33].

## Discussion

Our fMRI study aimed to explore the neural basis underlying the retrieval of action representations and the execution of simple actions by examining finger-tapping sequences at different category levels. Finger-tapping tasks are commonly used in functional neuroimaging literature to investigate the human motor system, making them an ideal tool for studying the basis of representational and action execution processes (Witt et al. 2008). While employing simple motor movements, our experimental design featured the implementation of different action categories, which allowed us to provide participants with motor contexts to modulate the information accessible via a specific stimulus. Using a classification system that ranged from the most specific to the most general action categories, we could examine representational and action execution processes in a gradient fashion rather than focusing solely on motor-specific processes. In this regard, we hypothesized that the pBA44 area would play a key role in retrieving action representations through the information available from the context. At the same time, BA6 would be responsible for integrating information from various trial phases into the motor plan and transforming the representation into action.

**Fig. 7:**
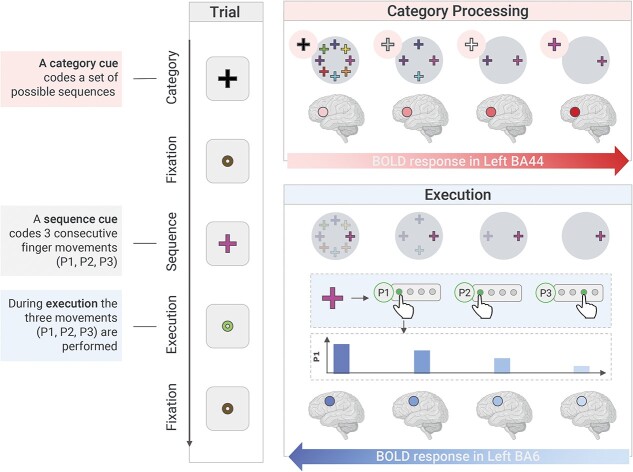
Summary of the experimental design and results. On the left, we provide an example trial for the General level of the variable Category. Every trial is structured as follows: First, a Category cue is presented (Category Processing phase). This cue codes a set of possible sequences from which the sequence to be executed will be selected. This is followed by a fixation cue and then by a sequence cue, which signals the sequence to be executed (e.g. the pink cross stands for the sequence 1-1-3). When the green circle appears (Execution phase), the participant makes three presses (Ps) to execute the sequence: in this case, P1 = 1, P2 = 1, P3 = 3. Lastly, a fixation circle appears again on the screen. Our main findings show that during the Category processing phase (in red), there is an increase in BOLD response within BA44, which corresponded with more specific action categories. Conversely, during the Execution phase (in blue), we found an increase in BOLD response within BA6, which corresponded with less specific action categories. Behaviorally, we observed that the mean time to make P1 also increased with less specific action categories. Brain icons were created with BioRender.com.

To investigate the distinct cortical responses related to the retrieval of action representations and action execution, we employed a delayed-movement paradigm in which participants first processed the action category (Category Processing phase) and then executed an action from that category (Execution phase). This made it possible to identify brain regions responding incrementally or decrementally to the level of the Category presented. We found two distinct patterns when testing for regions at the whole-brain level showing a linearly decreasing or increasing response pattern during the Category Processing or Execution phases, respectively. First, we observed that during the Category Processing phase, activity in pBA44 linearly decreased as less specific action categories were being processed. Second, activity in the BA6 during the Execution phase increased linearly as more general action categories were being processed. Overall, these findings help us understand how representations are translated into actions, highlighting the functional relevance of pBA44 and BA6 in this process (for a schematization of the experimental design and of the main findings, see Fig. 7).

### Left pBA44 in category processing

Previous research has identified left BA44 as a crucial region in a wide range of cognitive tasks that require integrating information from multiple sources, such as language and perception, or coordinating different action plans (Koechlin and Jubault 2006). BA44 has also been implicated in selecting premotor representations based on the information provided by contextual signals of the type of stimulus–response associations (Koechlin et al. 2003; Koechlin and Summerfield 2007), indicating its relevance in motor-related processes. In addition, research has shown that BA44 is recruited whenever participants are required to switch between tasks or perform multiple tasks simultaneously (Koechlin and Jubault 2006), suggesting its role in coordinating different action plans to adapt behavior in response to changing task demands or goals.

Here we go beyond previous findings, providing evidence concerning the localization of the response and function of BA44 within the motor domain. Specifically, our results identify a posterior sub-region of BA44 (pBA44) involved in processing information in mental representations of actions. In addition, we show how this process is relevant to the premotor cortex for correctly executing the content of any representation, i.e. to transform a mental representation into an action performed by an agent. While previous studies associated BA44 with selecting mental representations of actions, they assumed that the area would be active exclusively if a perceived stimulus could provide complete motor information for future behavior—i.e. one-to-one mapping between an external stimulus and an action (e.g. when I hear my phone’s ringtone, the ringtone signals I have to answer). In our experiment, we show that pBA44 is instead processing any available information following a gradient of activation. The more information is available, the more pBA44 will be engaged in the selection process. The presence of an information-based modulation in pBA44 challenges previous claims that the area is responsible for encoding and storing motor sequences as single components (Clerget et al. 2011) or for encoding action–goal relationships (Fazio et al. 2009).

If BA44 encoded motor sequences as single components, one could have expected (i) activity within the area only in those cases in which the sequence was fully provided (Specific level) or (ii) a gradient of activation increasing towards less specific action Categories, as they encode more sequences simultaneously. This was, however, not the case since our results revealed a gradient of activation within BA44 increasing with more specific action Categories, suggesting that the area processes actions as a set of representational features to guide behavior. Additionally, we believe our results not to be influenced by goal-directed processes, as there are no goal differences across Category levels.

A supplementary finding of this study is that our results appear to reinforce the idea that BA44 is not a functional monolith but can be parcellated into functional sub-regions (Clos et al. 2013), as we observed that the cluster identifiable as BA44 in our functional contrast largely overlaps with posterior portions of the region. This localization aligns with a recent meta-analysis on motor domains, where we localized convergence for action execution, imitation, and imagery in pBA44 (Papitto et al. 2020).

### Left BA6 in execution

The premotor cortex has been acknowledged as a critical region in processing sensorimotor representations (Caminiti et al. 2017). It has been suggested that activity within the premotor cortex reflects the information contained in sensory stimuli regarding the nature and metrics of potential future actions (Wise et al. 1992). This idea was supported by a study in which monkeys were presented with two opposing potential-reaching actions, but only one would later be selected. The authors found that only the representation of the chosen action was strengthened in the premotor cortex while the unwanted action was suppressed (Cisek and Kalaska 2005). We found that the premotor cortex not only strengthens sensory-motor representations relevant to behavior but also adjusts its response to the specificity of the context conveyed by a category. Our results show that the less specific the context is, the more activity in BA6. This pattern is coherent with the view that the premotor cortex plays a central role in coordinating different types of motor and nonmotor information generated in the prefrontal and parietal cortices to convert the chosen action representation into a physical movement (Tanji and Hoshi 2001; Bunge et al. 2005). As such, the role of the premotor cortex is not limited to the pure motoric buffer. Instead, it combines past experience and sensory information to deduce unseen variables and adjust sensory perceptions to facilitate behavior (Qi et al. 2022).

Although previous research has shown that the neural activity in the premotor cortex is connected to the kinematics of hand movements, such as position, speed, and direction (Churchland et al. 2006), or to increased movement complexity (Lewis et al. 2004), we ruled out the interference of these factors in our experiment. Hand metrics were kept identical across Category levels, and motor specifics were balanced across participants (e.g. the white and gray cues coded different sequences for different participant groups). Therefore, our results correlate with the amount of information a particular stimulus carries and how this information informs future behavior.

This view is further supported by the behavioral data we collected in the pilot and the fMRI experiment. Reaction time data provided an accurate view of the underlying processes involved in the experimental manipulation. Participants had to execute finger-tapping sequences of three movements. Reaction time data were analyzed accordingly for the three movements: (i) P1 captures the time needed to translate the motor plan into the execution of the first movement; (ii) P2 captures the time needed to execute the second movement; and (iii) P3 captures the time needed to execute the third movement. While P2 and P3 capture performance once the motor plan has been formed and implemented, P1 provides information on the plan-to-execution processes (Botwinick and Thompson 1966; Westerholz et al. 2013). Consistent with our hypothesis, we observed that only P1 values were significantly affected by the experimental manipulation. We observed a significant increase in P1 mean values from Specific to Sub-Rule, Sub-Rule to Rule, and Rule to General. On the contrary, employing a Bayesian framework, we observed that the null hypothesis better explained P2 and P3 values, indicating that once the representation had been put into action, action performance was not affected by the specificity of the category levels. These results suggest that the gradient-like variation in neural activity observed in the premotor cortex is related to the representational properties of action categories rather than a variation of motor complexity across the different levels of the Category condition.

### Comparing left BA44 and BA6 responses across phases

While it is relevant to address phase-to-region mappings (i.e. pBA44’s role in Category processing, BA6’s role in Execution processing), further insights into pBA44 and BA6 contributions to action-related processes come from studying how both cortical areas are affected in both phases of the task. An exploratory signal change analysis revealed distinct behaviors for the two regions in the two phases. One cautionary note concerns signal change’s linearity in BA44 during the Category Processing phase and signal change’s linearity in BA6 during the Execution phase. These two analyses are performed only for illustrative and confirmatory purposes as they pose a dependence problem that makes their interpretation limited. This is due to the ROIs being generated on phase-specific linear responses at the GLM level and then used for testing for the signal change of the same phase.

During the Category Processing phase, similar to pBA44, BA6 percent signal change linearly increased with more specific categories. This response pattern supports the idea that the premotor cortex integrates available information along the task to process sensorimotor representations (Caminiti et al. 2017; Qi et al. 2022). Our results also suggest that BA6 may process and store information for later stages, consistently with the idea that the region retrieves and combines motor-related information with other relevant information (Hoshi 2007). Specifically, if all the information required to guide future behavior is available at the beginning of the trial (Specific level), BA6 may not need to retrieve it again during Execution. However, if only partial (Sub-Rule and Rule levels) or no information (General level) is accessible at the beginning of the trial, BA6 will need to retrieve this information later on. This is consistent with BA6’s response pattern observed during the Execution phase (see section Left BA6 in execution), where the percent signal change in the area linearly increased as more general categories were processed. We are cautious in interpretating these results as we did not generate explicit hypothesis concerning the involvement of BA6 during the Category Processing phase.

Although pBA44 is modulated linearly during the Category Processing phase, we did not observe any linear modulation of the percent signal change during the Execution phase. This might suggest that BA44 retrieves context-specific information to be sent to the premotor cortex. From a representational standpoint, the information provided during the Execution phase did not differ across the various Category levels, while it differed in the Category Processing phase. During the Category Processing phase, participants were presented with a cue linked to a category that included varying degrees of information. In contrast, during the Execution phase, participants were presented with cues always containing complete motor information. As a result, we suggest no differences could be observed in BA44 regarding the Execution phase, as no modulation of the Category levels was implemented at that stage.

### Further brain regions involved in category processing

Extended bilateral clusters spanned the postcentral gyrus (somatosensory regions BA2, BA3), which has been suggested to play a role in cognitive control processes (Smith et al. 2004; Wu et al. 2013, 2021). For instance, it was recently found that an increase in activation in the postcentral gyrus is associated with increases in information entropy, which suggests a mediation role of this cortical cluster, coordinating the amount of information available and behavioral performance (Wu et al. 2021). Other regions of bilateral activation are the inferior parietal lobule (BA40) and BA9 in the IFG. BA40 was shown to be involved in computing and strengthening sensory-motor associations and generating motor intentions (Mattingley et al. 1998; Meltzoff and Decety 2003). Thus, its increasing response with more specific levels might relate to the creation of motor plans corresponding to the motor information provided. In contrast, the activation of BA9 might result from the sustained attention required for the task (MacDonald et al. 2000). Additionally, we found right-hemispheric BA45 and BA47 activations within the PFC. These regions are associated with response inhibition processes (Sakagami and Niki 1994; Blasi et al. 2006), which our experiment required as participants had to retrieve information relevant to action execution but waited for a second cue before performing the finger-tapping task, and they had to select appropriate action representations of a particular category while suppressing competing representations from higher-level categories. A further cortical cluster spanned right BA7, which has been found to be engaged during memory retrieval, suggesting that activity in the cluster is related to the memory retrieval of available motor information and its execution (Sakai et al. 1998; Shannon and Buckner 2004; Witt et al. 2008; Koenigs et al. 2009).

### Further brain regions involved in execution

Cortical activations exclusively attributable to the Execution phase spanned left-hemispheric BA45 and BA7. Previous research has shown that BA45 plays a role in actively retrieving information from memory, when the information stored cannot be easily retrieved through the presence of a specific stimulus or context (Petrides 2002, 2005). The result of increased activity in BA45 with less specific action categories is consistent with the latter findings, and it suggests that the area may be involved in less automatic information retrieval processes. Similarly, BA7 has been implicated in the retrieval and later execution of memorized (action) sequences, as discussed in the previous section.

### The role of the insula in category processing and execution phases

The insula was the only region we found symmetrically modulated by Category levels during the Category Processing and Execution phases. When presented with increasing category specificity, insular activity increased during the Category Processing phase and decreased during Execution. The insula has been proposed to be responsible for maintaining task sets and controlling behavior by integrating different bits of information (Dosenbach et al. 2008). In line with this idea, employing a perceptual decision-making task with increasing cognitive demands, it was shown that activity in the insula increased linearly with the amount of information provided (Wu et al. 2019). Our study was also able to show a similar pattern during the Category Processing phase, but we observed the opposite pattern during the Execution phase, where the less specific the category provided, the less insular activity. Granted that insular activation is sustained through task epochs, we suggest that during the Execution phase, information from different trial phases is integrated by the area (e.g. if no or little information is provided to the area in the Category Processing phase, complete motor information has to be retrieved during Execution).

### Future directions

While this study provides valuable insights into the neural mechanisms underlying action category processing, several future directions should be undertaken. Firstly, although the categorization into Category levels was reliable at the behavioral level, we recognize that this task does not fully capture the overall complexity of real-world scenarios where many competing actions are present simultaneously. Future studies could use richer action categories to reflect the complexity of human contexts of behavior. Secondly, although the finger-tapping task was practical in probing cognitive processes, it is essential to note that it is a relatively simple motor task. Therefore, the generalization of these findings to more structured actions may be limited. Thirdly, employing an action execution task limits the possibility of generalizing these findings to action observation or motor imagery. Future studies might address whether and how the processes we investigated map to other forms of action processing. Lastly, our delayed-movement paradigm does not allow to correlate pBA44’s response to behavior, as it is impossible to determine whether slower RTs or lower accuracy rates are to be attributed to processing the Category or the Sequence cue, or to their integration. Further studies should introduce intermediate behavioral measure to directly investigate to what extent the category has been processed, before the to-be-performed sequence is shown. This will provide the possibility to better correlate cognitive control and execution processes.

## Conclusion

Our fMRI study provides novel insights into the neural mechanisms underlying action processing. By employing a delayed-movement paradigm and a classification system ranging from specific to general action categories, we could examine action representation access and action execution in a gradient fashion, revealing the distinct roles of pBA44 and BA6 in accessing representations and performing single actions out of them. Our findings suggest that pBA44 is modulated by action selection based on the motor information available from the context (as provided by the action categories). At the same time, BA6 integrates information from various task phases into the motor plan and transforms the representation into action. Overall, these results contribute to understanding how representations are translated into actions and provide new evidence on how pBA44 orchestrates behavior and how BA6 supports motor action execution, shedding light on the fundamental cortical mechanisms underlying human behavior.

## Supplementary Material

Supplementary_Materials_bhae163
