# Supplementary material for: Distinct neural mechanisms for action access and execution in the human brain: insights from an fMRI study
Source: Cereb Cortex. 2024 Apr 14;34(4):bhae163. doi: 10.1093/cercor/bhae163 (PMC11022341; doi:10.1093/cercor/bhae163)
Supplement: Supplementary_Materials_bhae163 [file supplementary_materials_bhae163.pdf]

Supplementary Material

Index

1. Behavioral pilot **2**

1.1 Experimental procedure *2*

1.1.1 Participants 2

1.1.2 Pilot experimental paradigm 2

1.1.3 Behavioral data analysis 2

1.2 Pilot experiment: behavioral results *3*

1.2.1 Press 1 (P1) 3

1.2.2 Press 2 (P2) 3

1.2.3 Press 3 (P3) 3

2. Supplementary Tables **5**

2.1 Supplementary Table 1 .... *5*

2.2 Supplementary Table 2 .... *7*

2.3 Supplementary Table 3 .... *8*

References **9**

1. Behavioral pilot

The experimental paradigm was behaviorally piloted to ensure that participants could learn eight finger-tapping sequences and the relationships across categories with only one day of training. In the behavioral pilot, participants underwent a training session, composed of phases (TP1 and TP2; Section 2.2.2) and an experimental session outside the scanner.

1.1 Experimental procedure

1.1.1 Participants

Data from twenty native German speakers (12 female; mean age = 23.3 years; Standard Deviation (SD) = 3.26; range = 19–30) were analyzed. Participants that took part in the pilot experiment did not take part in the fMRI experiment. Each participant took part in one training session and one experimental session. Twenty-four participants were initially recruited, but four performed poorly during the training session and were excluded from the experimental session. All participants were right-handed (mean laterality quotient = 90.37; SD = 14.66), as assessed with the Edinburgh handedness test (Oldfield, 1971), and had no history of neurological disorders. They were recruited via the participant database of the Max Planck Institute for Human Cognitive and Brain Sciences, Leipzig, Germany. Written informed consent was obtained from each participant before the experiment. The study was performed according to the guidelines of the Declaration of Helsinki, and it was approved by the local ethics committee of the University of Leipzig. Participants were reimbursed 9€ per hour for participating in the study. Moreover, each participant could receive a little additional monetary compensation, following the same criteria of the functional Magnetic Resonance Imaging (fMRI) experiment.

1.1.2 Pilot experimental paradigm

To a great extent, the behavioral pilot paradigm matched the experimental paradigm we employed for the fMRI experiment. Therefore, this section focuses exclusively on the pilot experiment features later changed in the fMRI experiment. First, the color of the fixation cues in the pilot's training and experimental sessions was white, while the color of the Execution cue was light green. Second, the cue at the end of each block of the experimental session was a red circle. We later changed the colors so that these cues would not have the same colors as the Sequence or Category cues. Using similar colors could have affected the Sequence or Category cues' processing. Third, the time to complete the finger-tapping sequence during the experimental session was 2500ms. As previously specified, this was later reduced to 2000ms to match the average execution times of participants. Lastly, at the end of both sessions, we presented participants with a small questionnaire to get feedback on the experiment. In the behavioral pilot experiment, the training and experimental sessions took place in the same laboratory, with precisely identical settings as TP1 and TP2 in the fMRI experiment.

1.1.3 Behavioral data analysis

Pilot data were preprocessed and analyzed following the same steps described for the behavioral analysis of the fMRI session (Section 2.5).

1.2 Pilot experiment: behavioral results

Participants were generally accurate in performing the delayed-movement task in the pilot experiment (mean = 97.719%; SD = 1.537). Logistic regression analysis results show a main effect of Category on accuracy rates ($\chi^2_3 = 15.27$; $p < .01$), with more specific Categories leading to higher accuracy rates. Mean accuracy was calculated for each level of the variable Category: *Specific* (mean = 97.604%; SD = 2.890), *Sub-Rule* (mean = 99.167%; SD = 1.246), *Rule* (mean = 97.813%; SD = 2.387), *General* (mean = 96.771%; SD = 2.572). Here below, we report the results of frequentist and Bayesian analyses for each finger-tapping movement.

1.2.1 Press 1 (P1)

A frequentist one-way ANOVA on mean P1 values resulted in a main effect of condition ($F_{(3,76)}$ = 26.53; $p < .001$). Similar results were obtained when employing a Bayesian ANOVA, which attested robust evidence for rejecting the null hypothesis (BF_{10} = 1.038+e9). To check for significant increases in P1 from one Category level to the other, we ran three one-sided paired-sample *t*-tests (Bonferroni corrected for three comparisons) as follows: (1) *Specific* < *Sub-Rule*; (2) *Sub-Rule* < *Rule*; (3) *Rule* < *General*. Respectively, we observed that: (1) P1 values relative to the *Specific* level are reduced (mean = .436; SD = .083) when compared to those of the *Sub-Rule* level (mean = .471; SD = .0939; $t_{(19)}$ = -3.508; p = .004); (2) P1 values relative to the *Sub-Rule* level are reduced when compared to those of the *Rule* level (mean = .570; SD = .104; $t_{(19)}$ = -9.787; $p < .001$); and (3) P1 values relative to the *Rule* level are reduced when compared to those of the *General* level (mean = .710; SD = .137; $t_{(19)}$ = -8.783; $p < .001$). Paired-sample post-hoc comparisons were also performed using a Bayesian approach. In this case, we also observed: (1) positive evidence in favor of the alternative hypothesis when testing for *Specific* < *Sub-Rule* (BF_{-0} = 34.828); (2) robust evidence when testing for *Sub-Rule* < *Rule* (BF_{-0} = 3.299+e06); and (3) robust evidence when testing for *Rule* < *General* (BF_{-0} = 681718.421). Finally, a linear trend analysis revealed that P1 values were associated with a significant positive linear trend ($F_{(1, 19)} = 165.67$; $p < .001$; η^2_p =.90).

1.2.2 Press 2 (P2)

A frequentist one-way ANOVA on mean P2 values resulted in no significant main effect of Category ($F_{(3,76)}$ = .137; p = .938). While the classical frequentist ANOVA rejected H_1, a Bayesian ANOVA provided weak evidence in favor of H_0 (BF_{01} = 12.640). The following mean values for each Category level were observed: (1) *Specific* (mean = .268; SD = .071); (2) *Sub-Rule* (mean = .266; SD = .067); (3) *Rule* (mean = .258; SD = .061); (4) *General* (mean = .257; SD = .063). Finally, a linear trend analysis revealed that P2 values were associated with a significant negative linear trend ($F_{(1, 19)}$ = 16.48; $p < .05$; η^2_p =.46).

1.2.3 Press 3 (P3)

P3 data were normalized using a Box-Cox transformation since a D'Agostino-Pearson test on mean values reported a significant deviation from normality (p = .005). A frequentist one-way ANOVA on normalized mean P3 values resulted in no significant main effect of Category ($F_{(3,76)}$ = .136; p = .938). While the classical frequentist ANOVA rejected H_1, a Bayesian ANOVA provided weak evidence in favor of H_0 (BF_{01} = 12.653). The following mean values for each Category level were observed: (1) *Specific* (mean = -1.159; SD = .207); (2) *Sub-Rule* (mean = -1.190; SD = .197); (3) *Rule* (mean = -

1.188; SD = .203); (4) *General* (mean = -1.95; SD = .193). Finally, a linear trend analysis revealed that P3 values were associated with a significant negative linear trend ($F_{(1, 19)}$ = 18.8; p < .01; η^2_p =.49).

2. Supplementary Tables

2.1 Supplementary Table 1

Region	BA	Hemisphere	Cluster size	MNI coordinates			T
				x	y	z	
SupramargGyr	BA40	L	749	-54	-25	43	10.88
VisMotor	BA7	L		-39	-43	52	10.80
PrimSensory	BA1	L		-45	-31	52	9.82
PreMot SuppMot	BA6	L	159	-57	8	31	10.08
PreMot SuppMot	BA6	L		-45	-1	10	6.66
PreMot SuppMot	BA6	L	107	-3	-1	55	8.71
SupramargGyr	BA40	R	352	42	-40	52	8.64
SupramargGyr	BA40	R		48	-31	46	8.03
AngGyrus	BA39	R		33	-61	49	6.77
Culmen		R	133	27	-55	-23	8.14
Culmen		R		30	-49	-29	7.54
Culmen		R		39	-43	-32	5.77
PreMot SuppMot	BA6	R	89	27	-4	55	7.87
PreMot SuppMot	BA6	R		36	5	52	5.94
PreMot SuppMot	BA6	R	112	48	8	34	7.66
Broca Operc	BA44	R		57	11	22	5.86
Cerebellum		R	52	15	-64	-47	7.27
Thalamus		L	23	-15	-22	13	6.29
Thalamus		L		-9	-16	7	5.62

ParsOrbitalis	BA47	R	13	33	29	-2	5.92
Putamen		L	8	-24	2	4	5.55
Insula		L	7	-30	20	4	5.52
dlPFC(dorsal)	BA9	R	1	33	35	28	5.39
dlPFC(dorsal)	BA9	L	2	-33	35	31	5.36
FrontEyeFields	BA8	R	3	6	20	55	5.31
VisMotor	BA7	L	4	-15	-64	58	5.18
Cerebellum		L	1	-24	-67	-29	5.13
VisMotor	BA7	L	1	-12	-73	49	5.12

Regions showing a negative linear response during the Category Processing phase. Activation peaks for brain regions that exhibited a negative linear response during the Category Processing phase (parametric *t*-contrast: "+3": Specific; "+1": Sub-Rule; "-1": Rule; "-3). Results are obtained after the FWE $p < .05$ correction at the voxel level. BA = Brodmann Area, L = left, R = right.

2.2 Supplementary Table 2

Region	BA	Hemisphere	Cluster size	MNI coordinates			T
				x	y	z	
Insula		L	3	-30	26	1	5.68
VisMotor	BA7	L	10	-30	-58	49	5.55
Broca-Triang	BA45	R	4	33	29	1	5.52
PreMot SuppMot	BA6	L	3	-24	2	55	5.14

Regions showing a positive linear response during the Execution phase. Activation peaks for brain regions that exhibited a positive linear response during the Execution phase (parametric *t*-contrast: "-3": Specific; "-1": Sub-Rule; "+1": Rule; "+3"). Results are obtained after the FWE $p < .05$ correction at the voxel level. BA = Brodmann Area, L = left, R = right.

2.3 Supplementary Table 3

Cluster (C)	Number of Voxels	Overlapping Voxels	Total overlap (%)	Relative overlap (%)
C1	1604	736	27.19	46.7
C2	1366	278	10.27	17.64
C3	1950	36	1.33	2.28
C4	1321	321	11.86	20.37
C5	1376	205	7.57	13.01

Mass overlap analysis. Overlap between each cluster (Cs) of BA44 (Clos et al., 2013) and the BA44-spanning cluster observed for the [+3 +1 -1 -3] contrast at the whole brain level, as the region of interest (ROI). In columns, we report (1) the total number of voxels of each cluster; (2) the number of voxels overlapping between each cluster and the ROI; (3) the total overlap (%) between each cluster and the ROI; (4) the relative overlap between the ROI and each cluster, computed by dividing the number of voxels overlapping between a specific cluster-ROI combination (e.g., C1∩ROI) by the sum of the voxels overlapping for each C∩ROI combination (C1∩ROI + C2∩ROI + C3∩ROI + C4∩ROI + C5∩ROI), multiplied by 100.

References

Clos, M., Amunts, K., Laird, A. R., Fox, P. T., & Eickhoff, S. B. (2013). Tackling the multifunctional nature of Broca's region meta-analytically: Co-activation-based parcellation of area 44. *NeuroImage*, *83*, 174–188. https://doi.org/10.1016/j.neuroimage.2013.06.041

Oldfield, R. C. (1971). The assessment and analysis of handedness: The Edinburgh inventory. *Neuropsychologia*, *9*(1), 97–113. https://doi.org/10.1016/0028-3932(71)90067-4
